# Transcription Factor CTCFL Promotes Cell Proliferation, Migration, and Invasion in Gastric Cancer via Activating DPPA2

**DOI:** 10.1155/2021/9097931

**Published:** 2021-10-19

**Authors:** Haibo Yao, Qinshu Shao, Yanfei Shao

**Affiliations:** ^1^Department of Gastrointestinal and Pancreatic Surgery, Zhejiang Provincial People's Hospital (People's Hospital of Hangzhou Medical College, Key Laboratory of Gastroenterology of Zhejiang Province), 310014 Hangzhou, China; ^2^Department of Pharmacy, Zhejiang Provincial People's Hospital (People's Hospital of Hangzhou Medical College), 310014 Hangzhou, China

## Abstract

**Objective:**

To explore the relationship between CTCFL and DPPA2 and validate the positive role of CTCFL/DPPA2 in cell malignant behaviors in gastric cancer.

**Methods:**

We predicted gastric cancer-related transcription factors and corresponding target mRNAs through bioinformatics. Levels of CTCFL and DPPA2 were assessed via qRT-PCR and western blot. *In vitro* experiments were utilized to assay the cell biological behaviors. CHIP was utilized for the assessment of the targeted relationship between CTCFL and DPPA2.

**Results:**

CTCFL and DPPA2 were both highly expressed in gastric cancer cells, and high CTCFLL and DPPA2 could promote cell malignant behaviors. CHIP validated that DPPA2 was a target of CTCFL. In addition, high DPPA2 rescued the repressive impact of CTCFL silencing on the cell proliferation, migration, and invasion in gastric cancer.

**Conclusion:**

The transcription factor CTCFL fosters cell proliferative, migratory, and invasive properties via activating DPPA2 in gastric cancer.

## 1. Introduction

Gastric cancer is a gastrointestinal malignancy responsible for mortality worldwide [1]. Great advance has been achieved towards cancer treatment, whereas it is still a big challenge for gastric cancer treatment that metastasis occurs after the disease is radically cured [2]. Hence, it is necessary to perform in-depth research on the molecular mechanism underlying the migration and invasion of gastric cancer cells, as well as premise of cancer metastasis, so as to provide potential therapeutic strategies.

CCCTC-binding factor (CTCF) as a highly conserved protein exerts diverse functions on transcriptional regulation as well as chromatin architecture, and it can serve as a transcription factor mediating the insulation and cycling of chromatin; in short, CTCF is a necessity for life maintenance [3, 4]. The combination of CTCF with DNA sequences is predominantly realized via the 11-zinc finger region, which is beneficial for the protein-protein interactions. CTCFL is a homology of CTCF harboring a nearly identical 11-zinc finger region [5]. Meanwhile, these two proteins have similar binding specificity to DNA sequences due to the difference in the sequences on the amino and carboxyl terminals, but the protein functions are different [5]. In the current public literatures, CTCFL can mediate the onset and progression of varying cancers, such as liver cancer and neuroblastoma [6, 7], yet no relevant efforts have been made in gastric cancer.

DPPA2 is specifically expressed in pluripotent cells and some cancer tissues [8, 9]. It is involved in the pluripotent maintenance of embryonic stem cells and key to early embryogenesis and reprogramming of somatic cells into induced pluripotent stem cells [10–12]. DPPA2 is differentially expressed in diverse cancers and can be used as a specific therapeutic target in some tumors, such as ovarian cancer, colon cancer, lymphoma, and melanoma [13]. The original clinical studies reported that a high protein level of DPPA2 is implicated in lymphatic metastasis and further gastric cancer metastasis [14]. But the specific molecular mechanism by which DPPA2 affects gastric cancer progression is a highly unmet need.

We described differential CTCFL and DPPA2 expression in gastric cancer tissues. Meanwhile, we investigated the role of CTCFL/DPPA2 in cell proliferation, migration, and invasion and also validated the targeted relationship between CTCFL and DPPA2. In short, our study provides some references for target therapy for gastric cancer.

## 2. Materials and Methods

### 2.1. Bioinformatics Analysis

Gene expression files of STAD included in the TCGA (https://portal.gdc.cancer.gov/) database were accessed and then processed for gene ID transformation using the GTF (GRCh38.p5) files for getting the data of the mRNA expression profile. The profile contains 32 normal samples and 373 tissue samples of gastric cancer. The “edgeR” was used for identifying the differentially expressed mRNAs (DE mRNAs) with the critical value set to ∣logFC | >2 and adj. *p* value < 0.01. Afterwards, the sequences on the upstream 500 bp of the DE mRNAs were applied as putative promoter sequences, which were then used for the extraction of the DE transcription factors (TF) with the JASPAR database (http://jaspar.genereg.net/). The TFs were firstly subjected to FIMO software (http://meme-suite.org/tools/fimo) for predicting the target mRNAs and then processed for enrichment analysis in DE mRNAs (cor > 0.3, *p* < 0.05). The TFs with *q* value < 0.05 were identified as candidate TFs. Pearson correlation analysis was used for analyzing the correlation between the target TF and mRNA.

### 2.2. Clinical Samples

Human gastric cancer tissues (*n* = 15) and corresponding adjacent benign tissues (margin > 5 cm, *n* = 15) from June 2015 to June 2019 were procured from the Zhejiang Provincial People's Hospital with the approval of all subjects. All cancer samples were pathologically diagnosed and flash-frozen in liquid nitrogen and preserved at -80°C after being isolated. All subjects had never received any preoperative treatment, neither chemotherapy nor radiotherapy. Our study had been approved by the Ethic Committee of the Zhejiang Provincial People's Hospital.

### 2.3. Cell Culture

GES-1 (No.: CBP60512), a human normal gastric epithelial cell line, and AGS (No.: CBP60476), SGC-7901 (No.: CBP60500), HGC-27 (No.: CBP60480), and BGC-823 (No.: CBP60477), gastric cancer cell lines, were all purchased from the Cell Bank of the China Center for Type Culture Collection, Chinese Academy of Sciences (CTCC; Shanghai, China). All cells were cultured in Dulbecco's modified Eagle medium (DMEM; Thermo Fisher Scientific, Inc., USA) supplemented with 10% fetal bovine serum (FBS; Gibco, USA) and then maintained in a 37°C incubator containing 5% CO_2_.

### 2.4. Cell Transfection

Vectors oe-CTCFL, sh-CTCFL, oe-DPPA2, and sh-DPPA2 and their matched negative controls (oe-NC and sh-NC) were synthesized by GenePharma (Shanghai, China). Cells (1 × 10^5^) before transfection were firstly incubated in 12-well plates. The LipoFiter assay kit (Hanbio, Shanghai, China) was applied for conducting the transfection process per the manufacturer's protocols. At 48 h after transfection, total RNA and protein isolation was completed.

### 2.5. qRT-PCR

Total RNA was isolated from cells with TRIzol (Invitrogen, Carlsbad, USA) and then used for the synthesis of the cDNA with the reverse transcription assay kit (Invitrogen, Carlsbad, USA), following the standard process. ABI 7900HT instrument (Applied Biosystems, USA) with the miScript SYBR Green PCR Kit (Qiagen, Germany) was implemented for qRT-PCR under thermal cycling conditions: predenaturation at 95°C for 10 min, 40 cycles of 95°C for 5 s, 60°C for 30 s, and 72°C for 2 min. The results were normalized to the GAPDH level with the 2^-*ΔΔ*Ct^ method. The primers are designed as Table 1.

### 2.6. Western Blot

RIPA lysate buffer containing 1% protease inhibitor (Beyotime, Shanghai, China) was used for isolation of total proteins from cells, and the BCA protein assay kit (Beyotime, Shanghai, China) was applied for quantification. After being denatured at a high temperature, protein samples (30 *μ*g/pore) were electrophoresed by 10% SDS-PAGE and then transferred onto PVDF (Millipore) membranes. After being sealed with 5% skim milk for 2 h, membranes were incubated with primary rabbit polyclonal antibodies against CTCFL (ab126766, 1 : 1000; Abcam, China), DPPA2 (ab91318, 1 : 100; Abcam, China), and GAPDH (ab137321, 1 : 10000; Abcam, China) overnight at 4°C. On the following day, the secondary antibody horseradish peroxidase- (HRP-) labeled goat anti-rabbit IgG was added onto the membranes for hybridization at room temperature for 120 min and washed 3 times with 1x TBST (Solarbio, Beijing, China). After the reaction, the enhanced chemiluminescence (ECL) assay kit (Solarbio, Beijing, China) was employed for the visualization of the protein bands, and then, images were captured.

### 2.7. CCK-8

96-well plates were recommended for cell incubation (200 *μ*l, 1 × 10^4^ cells/ml). At 0, 24, 48, and 72 h, the reagent (20 *μ*g/well) supplied by the cell counting kit-8 (Yeasen) was added for 4 h of cell incubation at 37°C in 5% CO_2_. SpectraMax M5 (Molecular Devices, MD, USA) was recommended to measure absorbance values at 450 nm.

### 2.8. Wound Healing Assay

Cells (2 ml, 2.5 × 10^5^ cells/ml) were inoculated into 6-well plates until the confluence reached 90%. Then, the monolayer was wounded with the sterile pipette tip and sequentially washed with PBS and suspended by the FBS-free mediums under standard conditions. The wound areas at 0 and 48 h were photographed under an inverted microscope (40x).

### 2.9. Transwell Invasion Assay

Transwell inserts (Sigma, China) that were precoated with Matrigel matrix (BD, USA) were put into 24-well plates. 200 *μ*l of cells (1 × 10^5^ cells/ml) suspended by FBS-free mediums was planted into the inserts, and 10% FBS-supplemented mediums were added into the plates. At 24 h after incubation under routine conditions, cells that invaded the plates were exposed to 4% paraformaldehyde for fixation (30 min), followed by 0.1% crystal violet for staining (30 min). Cells still on the top of the membrane were softly swabbed with a wet cotton swab. Five fields of the view were randomly selected using an inverted microscope (100x) and then photographed for cell count.

### 2.10. Chromatin Immunoprecipitation- (ChIP-) PCR

The EZ-Magna ChIP assay kit (Millipore) was recommended. Specific procedures were as below: 1% formaldehyde solution was used to induce the cross-linking of cells, and 140 mM glycine was added for the reaction termination. After the cells were lysed, the nucleoprotein complexes were sheared to 200-500 bp, and then, the obtained DNA fragments were incubated with the antibody for immunoprecipitation overnight at 4°C. After being rinsed with 1x low salt buffer, 1x high salt buffer, 1x LiCl buffer, and 2x TE buffer, samples were sequentially eluted for 15 min at 37°C with 200 *μ*l of elution buffer. Thereafter, the samples were incubated with 5 M NaCl for the reversal of cross-linking overnight at 65°C and then treated with RNase and protease K. qRT-PCR was performed for identifying the combination of CTCFL and the DPPA2 promoter region.

### 2.11. Statistical Analysis

All data from three independent experiments were analyzed under the GraphPad Prism 7.0 software (GraphPad Software, Inc., La Jolla, CA). Measurement data were presented as the mean ± standard deviation. Comparisons between two groups and among multiple groups were analyzed by Student's *t*-test and one-way analysis of variance, respectively. *p* < 0.05 was set to be a threshold for statistical significance.

## 3. Results

### 3.1. Bioinformatics Analysis Results

**Figure 1:**
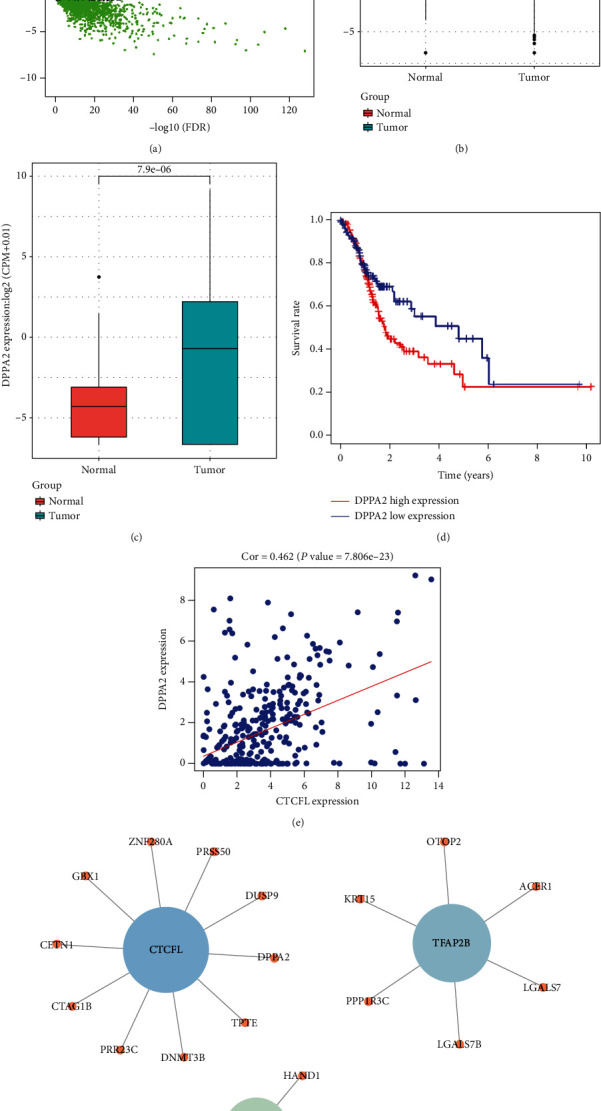
Bioinformatics analysis results. (a) DE mRNAs in the TCGA-STAD dataset identified by differential analysis. (b) CTCFL and (c) DPPA2 levels were tested in the TCGA-STAD dataset (red: normal; blue: tumor). Then, (d) Kaplan-Meier survival analysis was conducted on the DPPA2 in the TCGA-STAD dataset (red: high level; blue: low level), and (e) the relationship between the levels of CTCFL and DPPA2 was analyzed by Pearson correlation analysis. (f) The regulatory networks of CTCFL, TFAP2B, and SP8.

Totally, 1645 DE mRNAs (Figure 1(a)) and 62 DE TFs (Supplementary Table 1) were obtained. The DE TFs were used for the prediction of the target mRNAs using the FIMO software and then subjected to enrichment analysis in DE mRNAs. Among the DE TFs, 3 TFs with *q* value < 0.05 were identified, including TFAP2B, CTCFL, and SP8, and the mRNAs meeting cor > 0.3 and *p* < 0.05 were then projected onto corresponding TF regulatory networks (Figure 1(f)). CTCFL (BORIS) is a pivotal DNA binding protein involved in tumor regulation, and it also serves as a vital immunotherapeutic target [15, 16]. Besides, Pearson correlation analysis was conducted and found that there was a positive correlation between CTCFL and DPPA2 (Figure 1(e)). Hence, we selected CTCFL as our research object. Bioinformatics analysis revealed that CTCFL and DPPA2 were both upregulated in tumor tissues relative to the normal tissues in the TCGA-STAD dataset (Figures 1(b) and 1(c)). In addition, survival analysis suggested that CTCFL showed no marked correlation with patients' prognosis, while high DPPA2 was noticeably implicated in the unfavorable prognosis of patients (Supplementary Figure 1, Figure 1(d)). As DPPA2 is elevated in cancer cells and implicated with cell metastasis in gastric cancer [14], we reasoned that the TF CTCFL functions on cell malignant behaviors in gastric cancer via targeting DPPA2.

### 3.2. CTCFL and DPPA2 Are Upregulated in Gastric Cancer Tissue and Cells

**Figure 2:**
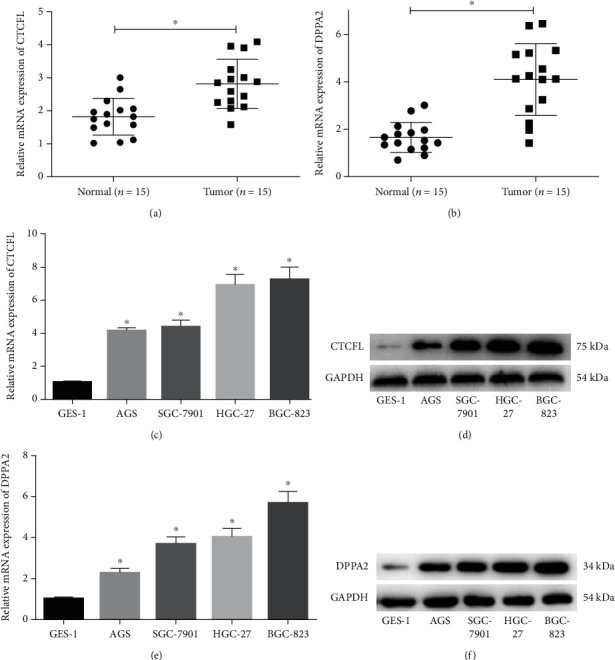
CTCFL and DPPA2 are highly expressed in gastric cancer tissue and cells. qRT-PCR and western blot displayed the mRNA level of (a) CTCFL and (b) DPPA2 in clinical tissue samples (tumor and adjacent) and displayed mRNA and protein levels of (c, d) CTCFL and (e, f) DPPA2 in GES-1, AGS, SGC-7901, HGC-27, and BGC-823 cell lines. ^∗^*p* < 0.05.

To be much clearer on the levels of CTCFL and DPPA2 in gastric cancer, clinical tissue samples (tumor and adjacent normal), GES-1, and 4 cancer cell lines were selected for further verification. qRT-PCR and western blot unveiled that CTCFL and DPPA2 were both significantly elevated in mRNA and protein levels in cancer cases relative to the corresponding controls (Figures 2(a)–2(f)), which showed a good consistence with the result of the above bioinformatics analysis.

### 3.3. Silencing CTCFL Inhibits Cell Malignant Behaviors in Gastric Cancer

**Figure 3:**
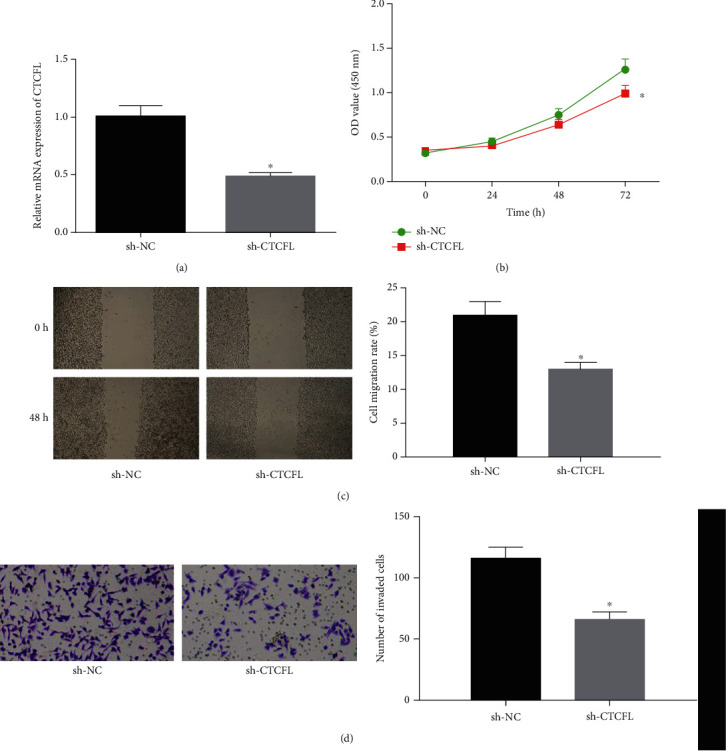
Silencing CTCFL hinders cell malignant behaviors in gastric cancer. sh-CTCFL and sh-NC were transfected into cancer cells. (a) qRT-PCR was conducted to test transfection efficiency. Then, the transfected cells were harvested for (b) CCK-8, (c) wound healing assay (40x), and (d) Transwell for determining cell biological behaviors (100x). ^∗^*p* < 0.05.

The CTCFL silencing cell line was generated by transfection of sh-CTCFL and sh-NC (Figure 3(a)). Then, CCK-8, wound healing assay, and Transwell invasion assay were performed to test the cell behaviors. As shown in Figures 3(b)–3(d), silencing CTCFL suppressed cell proliferative, migratory, and invasive properties. Collectively, CTCFL potentiated cell malignant behaviors in gastric cancer.

### 3.4. Silencing DPPA2 Hinders Cell Malignant Phenotypes in Gastric Cancer

**Figure 4:**
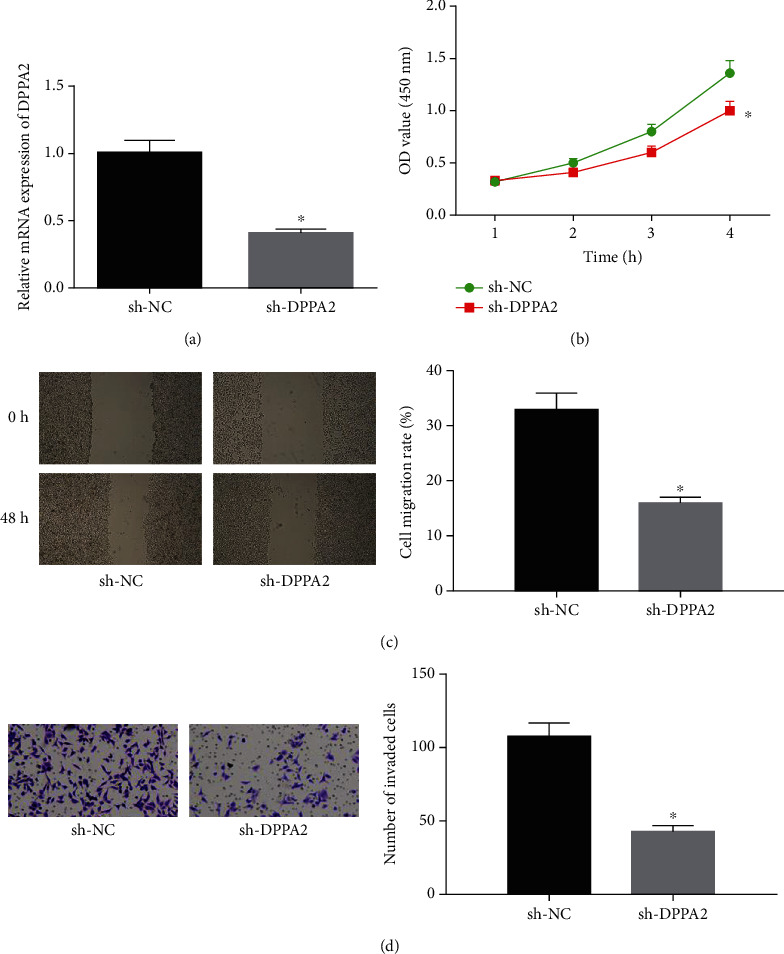
Silencing DPPA2 hinders cell malignant behaviors in gastric cancer. sh-DPPA2 and sh-NC were transfected into cancer cells. (a) qRT-PCR was conducted to test transfection efficiency. Then, the transfected cells were harvested for (b) CCK-8, (c) wound healing assay (40x), and (d) Transwell for determining cell biological behaviors (100x). ^∗^*p* < 0.05.

Similarly, DPPA2 was silenced for further investigation (Figure 4(a)). CCK-8 suggested that cell proliferation was significantly reduced in sh-DPPA2 transfected cells relative to the NC, and cell migration and invasion were as well decreased as evidenced by wound healing and Transwell assays (Figures 4(b)–4(d)). Taken together, it could be seen that DPPA2 played a promotive role in cell malignant phenotypes in gastric cancer.

### 3.5. CTCFL Positively Regulates the Expression of DPPA2

**Figure 5:**
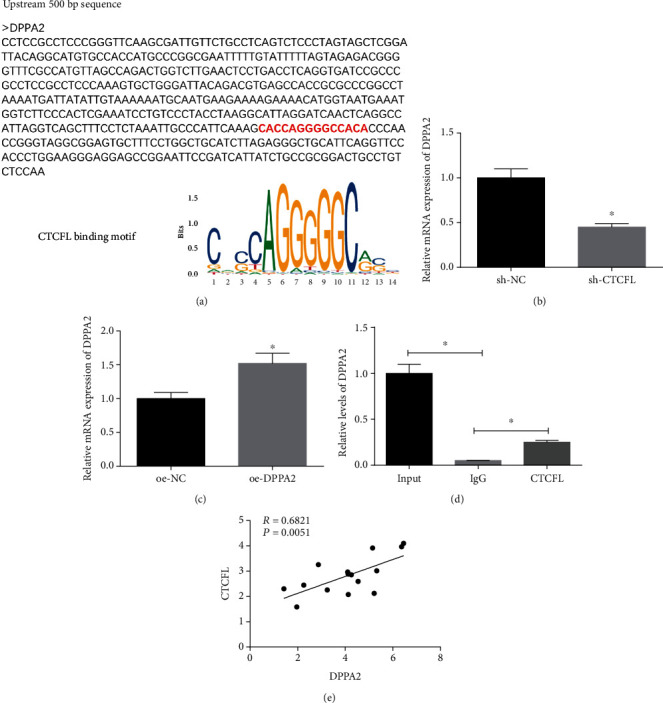
CTCFL promotes the DPPA2 level in gastric cancer. (a) Bioinformatics analysis was performed and discovered that there were potential binding sites of CTCFL on DPPA2 promoter. (b, c) qRT-PCR was carried out to determine the level of DPPA2 mRNA in cells transfected with (b) sh-CTCFL and (c) oe-CTCFL. (d) ChIP-PCR further validated the relationship between CTCFL and DPPA2, and (e) correlation analysis was performed on the levels of CTCFL and DPPA2 in the 15 gastric cancer tissue samples. ^∗^*p* < 0.05.

As abovementioned, CTCFL and DPPA2 both facilitated cell malignant behaviors in gastric cancer. Besides, potential binding sites of CTCFL on DPPA2 were predicted using the bioinformatics analysis (Figure 5(a)). To know more about the relationship between CTCFL and DPPA2, sh-CTCFL, oe-CTCFL, and matched NCs were transfected into cancer cells. Through qRT-PCR, CTCFL silencing was found to decrease the DPPA2 level (Figure 5(b)). Reversely, CTCFL overexpression increased the DPPA2 level (Figure 5(c)). In addition, ChIP-PCR was conducted for further verification of the interaction between CTCFL and DPPA2 promoter (Figure 5(d)). Moreover, correlation analysis indicated a positive correlation between CTCFL and DPPA2 (Figure 5(e)). Overall, these findings elucidated that DPPA2 was positively regulated by CTCFL.

### 3.6. The Repressive Effect of CTCFL Silencing on Cell Malignant Behaviors in Gastric Cancer Can Be Reversed by DPPA2 Overexpression

**Figure 6:**
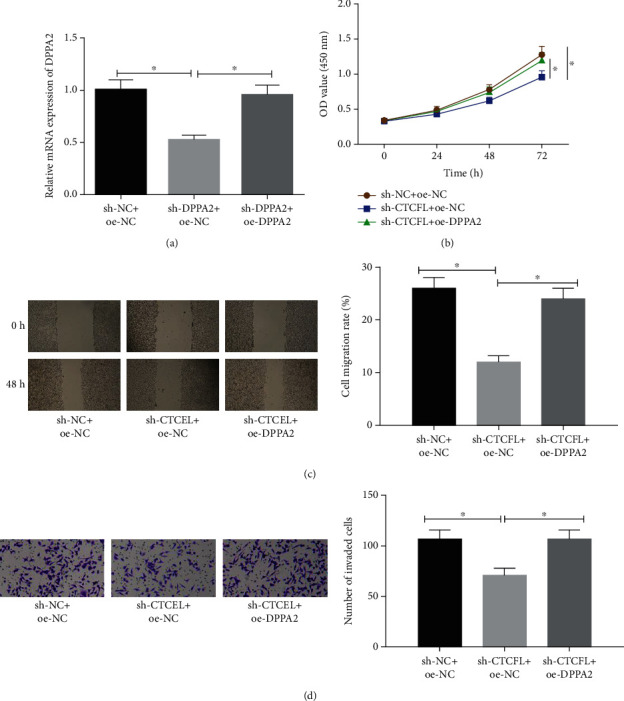
The repressive impact of CTCFL silencing on cell proliferation, migration, and invasion in gastric cancer can be reversed by DPPA2 overexpression. sh-NC+oe-NC, sh-CTCFL+oe-NC, and sh-CTCFL+oe-DPPA2 were transfected into cells. (a) qRT-PCR assessed transfection efficiency. Cells were collected for assessing the cell biological behaviors using the (b) CCK-8, (c) wound healing assay, and (d) Transwell invasion assay. ^∗^*p* < 0.05.

As we had confirmed that CTCFL could positively mediate DPPA2, to clearly clarify the underlying mechanism in gastric cancer, rescue experiments were further conducted. All cells were classified into 3 groups: sh-NC+oe-NC, sh-CTCFL+oe-NC, and sh-CTCFL+oe-DPPA2. qRT-PCR was performed for the assessment of the transfection efficiency (Figure 6(a)). Then, CCK-8 were conducted and showed that CTCFL silencing inhibited cell viability, but such inhibitory effect was attenuated when DPPA2 was simultaneously overexpressed (Figure 6(b)). Meanwhile, a similar result could be seen on cell migration and invasion as assessed by wound healing and Transwell assays (Figures 6(c) and 6(d)). Thus, we could conclude that DPPA2 overexpression suppressed the negative effect of CTCFL silencing on cell malignant phenotypes in gastric cancer.

## 4. Discussion

Transcription factors (TFs) are proteins that are able to bind with specific DNA sequences so as to ensure that their target genes can be expressed at a certain time and space with a certain intensity, and their dysfunction is the crucial pathological cause leading to the occurrence of malignant tumors [17]. For example, the TF E2F1 evokes the TINCR transcriptional activity and hastens gastric cancer progression through activating the TINCR/STAU1/CDKN2B axis [18]. The TF TFAP4 induces the PI3K/AKT pathway activation to potentiate cell metastasis in hepatocellular carcinoma (HCC) [19]. And the TF Nrf2 promotes the occurrence and development of bladder urothelial carcinoma by interacting with TUG1 [20]. CTCFL is intimately correlated with varying cancers. In HCC, for instance, CTCFL upregulates OCT4 by histone methylation to potentiate cancer stem cell-like properties [21]. And in breast cancer, CTCFL mediates the tumor occurrence and development in the way of inducing the activation of progesterone and estrogen receptor genes [22]. However, the role of CTCFL in gastric cancer, as well as the corresponding regulatory mechanisms, is unexplored.

We used bioinformatics analysis to know that CTCFL and DPPA2 were both differentially expressed in gastric cancer. To be more receivable, the levels of CTCFL and DPPA2 were assessed in clinical tumor tissues and matched adjacent benign tissues. It was found that CTCFL and DPPA2 were highly expressed in cancer cases, which demonstrated that these two genes might be implicated with the cell characteristics in gastric cancer. Subsequently, some *in vitro* experiments revealed that overexpressing CTCFL or DPPA2 promoted cell proliferation, migration, and invasion. It has been reported that CTCFL or DPPA2 exhibits a tight correlation with cell proliferation and metastasis in tumors. For example, DPPA2 knockdown exerts an inhibitory role in the proliferation of mouse stem cells [10] and is able to decrease the metastasis of cancer stem cells in neuroblastoma cell lines [7]. In view of these, we could see that overexpressing CTCFL or DPPA2 promotively functions on cell behaviors, but the regulation between themselves or towards gastric cancer is unclear.

Moreover, to further validate the targeted relationship between CTCFL and DPPA2, CTCFL silencing and overexpression cell lines were constructed. qRT-PCR was performed and found that the level of DPPA2 was positively altered with the level of CTCFL, showing that there was a certain relationship between the two genes. Hence, we conducted ChIP-PCR for further verification. As expected, CTCFL could target binding with DPPA2. Furthermore, rescue experiments were used to clarify the mechanism of CTCFL/DPPA2 in gastric cancer. The results revealed that overexpressing DPPA2 could attenuate the inhibitory effect of CTCFL silencing on cell behaviors. Collectively, in gastric cancer, TF CTCFL activates DPPA2 to foster cell proliferation, migration, and invasion.

In sum, this study finds that CTCFL and DPPA2 play an essential part in the malignant progression of gastric cancer, and their expression level may be associated with prognosis. Meanwhile, our study confirms the targeted relationship between DPPA2 and CTCFL, which may help to develop a novel strategy towards gastric cancer prevention and treatment. But the results were not confirmed by *in vivo* animal experiments. Further investigation will be done, including *in vivo* study and molecular mechanism of the CTCFL/DPPA2 axis in the disease.

## Figures and Tables

**Table 1:** qRT-PCR primer sequences.

Gene		Primer sequences
CTCFL	Forward	5′-AAAACCTTCCGTACGGTCACTCT-3′
	Reverse	5′-TGTTGCAGTCGTTACACTTGTAGG-3′

DPPA2	Forward	5′-AAGGAGGAGGAGGAGCCAAAC-3′
	Reverse	5′-TGGTTGGGTGTTTGATTCCAGC-3′

GAPDH	Forward	5′-TCCATGACAACTTTGGCATTG-3′
	Reverse	5′-CAGTCTTCTGGGTGGCAGTGA-3′

## Data Availability

The data and materials in the current study are available from the corresponding author on reasonable request.

## Supplementary Materials

Supplementary 1 Supplementary Figure 1: correlation between CTCFL level and patients' prognosis.

Supplementary 2 Supplementary Table 1: the 62 identified DE TFs.
