# Supplementary figures and images for: Transcription Factor CTCFL Promotes Cell Proliferation, Migration, and Invasion in Gastric Cancer via Activating DPPA2
Source: Comput Math Methods Med. 2021 Oct 19;2021:9097931. doi: 10.1155/2021/9097931 (PMC8548907; doi:10.1155/2021/9097931)

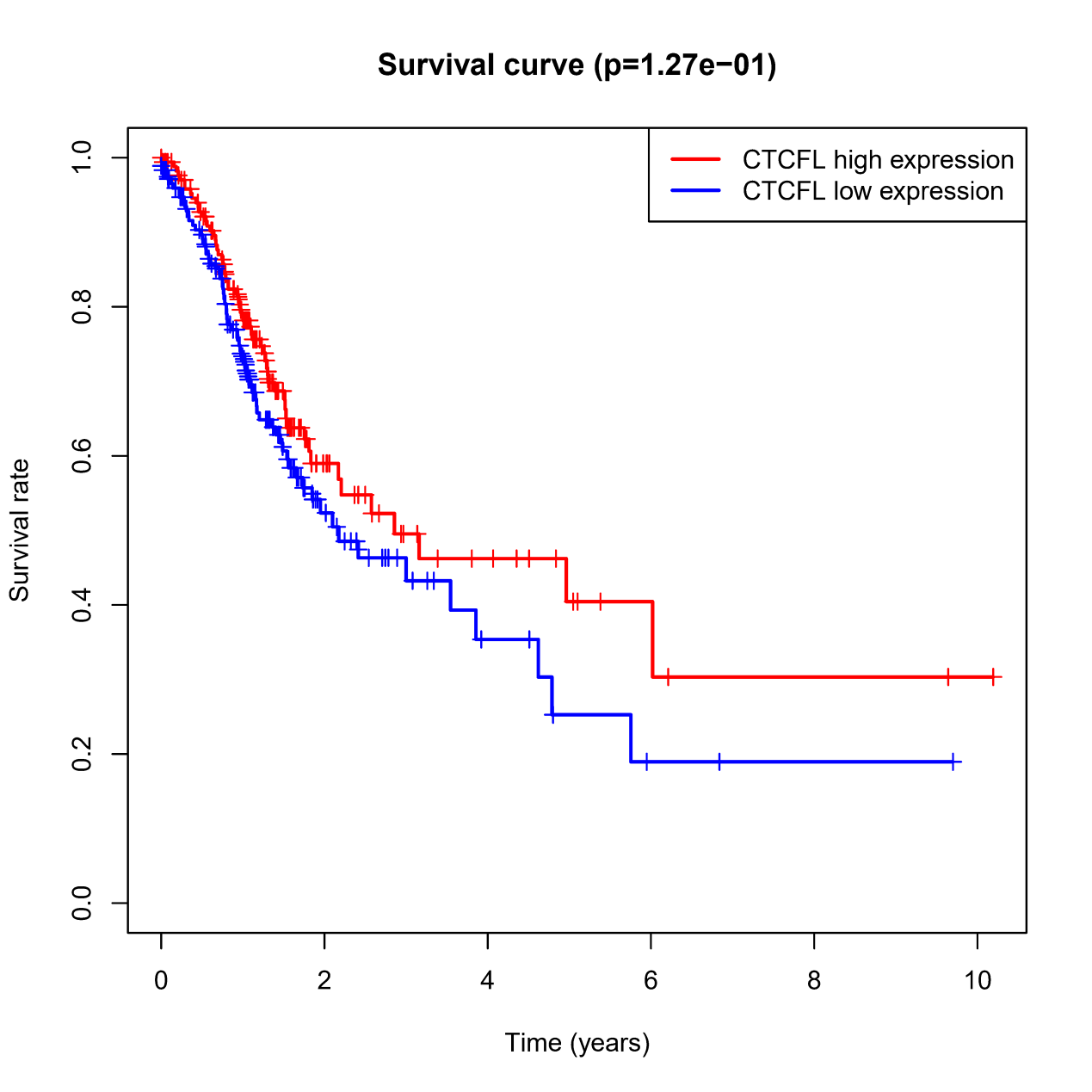

Supplement: Supplementary 1 — Supplementary Figure 1: correlation between CTCFL level and patients' prognosis. [file 9097931.f1.docx]
