# Supplementary material for: Transcription Factor CTCFL Promotes Cell Proliferation, Migration, and Invasion in Gastric Cancer via Activating DPPA2
Source: Comput Math Methods Med. 2021 Oct 19;2021:9097931. doi: 10.1155/2021/9097931 (PMC8548907; doi:10.1155/2021/9097931)
Supplement: Supplementary 2 — Supplementary Table 1: the 62 identified DE TFs. [file 9097931.f2.docx]

**Supplementary Table 1: The 62 identified DE TFs**

|  | total_genes | total_taget_genes | diff_genes | diff_targets | pvalue | q_value |
| --- | --- | --- | --- | --- | --- | --- |
| ELF5 | 58327 | 886 | 1645 | 22 | 0.757382 | 1 |
| HOXC12 | 58327 | 85 | 1645 | 1 | 0.91227 | 1 |
| SIX1 | 58327 | 556 | 1645 | 15 | 0.605323 | 1 |
| HOXC10 | 58327 | 327 | 1645 | 4 | 0.983209 | 1 |
| ESX1 | 58327 | 251 | 1645 | 5 | 0.838538 | 1 |
| PROX1 | 58327 | 238 | 1645 | 5 | 0.803657 | 1 |
| POU6F2 | 58327 | 432 | 1645 | 9 | 0.861261 | 1 |
| TFAP2B | 58327 | 1657 | 1645 | 79 | 5.42E-06 | 0.000325 |
| OLIG3 | 58327 | 236 | 1645 | 7 | 0.499529 | 1 |
| LHX9 | 58327 | 0 | 1645 | 0 | 1 | 1 |
| E2F7 | 58327 | 502 | 1645 | 16 | 0.344746 | 1 |
| BARX1 | 58327 | 0 | 1645 | 0 | 1 | 1 |
| HOXA10 | 58327 | 990 | 1645 | 22 | 0.896677 | 1 |
| SOX15 | 58327 | 559 | 1645 | 14 | 0.710485 | 1 |
| HLF | 58327 | 159 | 1645 | 3 | 0.82904 | 1 |
| LMX1B | 58327 | 0 | 1645 | 0 | 1 | 1 |
| ZIC4 | 58327 | 2309 | 1645 | 84 | 0.011225 | 0.662297 |
| HOXD12 | 58327 | 78 | 1645 | 1 | 0.892788 | 1 |
| DLX6 | 58327 | 0 | 1645 | 0 | 1 | 1 |
| ONECUT1 | 58327 | 448 | 1645 | 7 | 0.970172 | 1 |
| NKX6-2 | 58327 | 0 | 1645 | 0 | 1 | 1 |
| MYBL2 | 58327 | 76 | 1645 | 5 | 0.063652 | 1 |
| EN2 | 58327 | 243 | 1645 | 13 | 0.02138 | 1 |
| HOXD11 | 58327 | 75 | 1645 | 0 | 1 | 1 |
| GBX1 | 58327 | 252 | 1645 | 8 | 0.417496 | 1 |
| HOXD13 | 58327 | 640 | 1645 | 14 | 0.864927 | 1 |
| HOXC13 | 58327 | 141 | 1645 | 3 | 0.762986 | 1 |
| PAX3 | 58327 | 223 | 1645 | 2 | 0.987443 | 1 |
| GBX2 | 58327 | 302 | 1645 | 8 | 0.619954 | 1 |
| GSX1 | 58327 | 307 | 1645 | 11 | 0.251742 | 1 |
| POU4F1 | 58327 | 792 | 1645 | 18 | 0.853234 | 1 |
| CTCFL | 58327 | 5007 | 1645 | 213 | 1.08E-09 | 6.71E-08 |
| OTX2 | 58327 | 0 | 1645 | 0 | 1 | 1 |
| POU5F1B | 58327 | 635 | 1645 | 9 | 0.993376 | 1 |
| RXRG | 58327 | 890 | 1645 | 27 | 0.376773 | 1 |
| EN1 | 58327 | 0 | 1645 | 0 | 1 | 1 |
| FOXI1 | 58327 | 0 | 1645 | 0 | 1 | 1 |
| SCRT1 | 58327 | 604 | 1645 | 18 | 0.439357 | 1 |
| ETV4 | 58327 | 643 | 1645 | 16 | 0.728586 | 1 |
| POU3F2 | 58327 | 974 | 1645 | 15 | 0.996933 | 1 |
| VAX1 | 58327 | 0 | 1645 | 0 | 1 | 1 |
| SP8 | 58327 | 7232 | 1645 | 268 | 1.69E-06 | 0.000103 |
| NKX6-1 | 58327 | 0 | 1645 | 0 | 1 | 1 |
| FOXD2 | 58327 | 0 | 1645 | 0 | 1 | 1 |
| HOXC11 | 58327 | 110 | 1645 | 0 | 1 | 1 |
| ZIC1 | 58327 | 1993 | 1645 | 70 | 0.03691 | 1 |
| ASCL1 | 58327 | 3353 | 1645 | 113 | 0.029373 | 1 |
| EMX1 | 58327 | 273 | 1645 | 12 | 0.087943 | 1 |
| FOXH1 | 58327 | 542 | 1645 | 9 | 0.970051 | 1 |
| HOXA13 | 58327 | 811 | 1645 | 15 | 0.969784 | 1 |
| LBX1 | 58327 | 0 | 1645 | 0 | 1 | 1 |
| OTX1 | 58327 | 0 | 1645 | 0 | 1 | 1 |
| PAX9 | 58327 | 296 | 1645 | 8 | 0.597505 | 1 |
| RFX4 | 58327 | 937 | 1645 | 22 | 0.836471 | 1 |
| TP63 | 58327 | 248 | 1645 | 9 | 0.267987 | 1 |
| SRY | 58327 | 245 | 1645 | 3 | 0.970159 | 1 |
| EVX1 | 58327 | 151 | 1645 | 4 | 0.618943 | 1 |
| TBX4 | 58327 | 0 | 1645 | 0 | 1 | 1 |
| SOX21 | 58327 | 542 | 1645 | 11 | 0.899174 | 1 |
| PHOX2A | 58327 | 441 | 1645 | 7 | 0.966504 | 1 |
| PAX7 | 58327 | 223 | 1645 | 2 | 0.987443 | 1 |
| GCM1 | 58327 | 292 | 1645 | 8 | 0.582181 | 1 |
